# Does adjusting for recall in trend analysis affect coverage estimates for maternal and child health indicators? An analysis of DHS and MICS survey data

**DOI:** 10.3402/gha.v9.32408

**Published:** 2016-11-07

**Authors:** Nobubelo K. Ngandu, Samuel Manda, Donela Besada, Sarah Rohde, Nicholas P. Oliphant, Tanya Doherty

**Affiliations:** 1 Health Systems Research Unit, South African Medical Research Council, Cape Town, South Africa; 2 Biostatistics Research Unit, South African Medical Research Council, Pretoria, South Africa; 3 School of Mathematics, Statistics and Computer Science, University of KwaZulu-Natal, Durban, South Africa; 4 Programme Division, Health, UNICEF, New York, NY, USA; 5 School of Public Health, University of the Western Cape, Cape Town, South Africa

**Keywords:** population surveys, missing data, statistical considerations, indicator definitions

## Abstract

**Background:**

The Demographic and Health Surveys (DHS) and Multiple Indicator Cluster Surveys (MICS) are the major data sources in low- and middle-income countries (LMICs) for evaluating health service coverage. For certain maternal and child health (MCH) indicators, the two surveys use different recall periods: 5 years for DHS and 2 years for MICS.

**Objective:**

We explored whether the different recall periods for DHS and MICS affect coverage trend analyses as well as missing data and coverage estimates.

**Designs:**

We estimated coverage, using proportions with 95% confidence intervals, for four MCH indicators: intermittent preventive treatment of malaria in pregnancy, tetanus vaccination, early breastfeeding and postnatal care. Trends in coverage were compared using data from 1) standard 5-year DHS and 2-year MICS recall periods (unmatched) and 2) DHS restricted to 2-year recall to match the MICS 2-year recall periods (matched). Linear regression was used to explore the relationship between length of recall, missing data and coverage estimates.

**Results:**

Differences in coverage trends were observed between matched and unmatched data in 7 of 18 (39%) comparisons performed. The differences were in the direction of the trend over time, the slope of the coverage change or the significance levels. Consistent trends were seen in 11 of the 18 (61%) comparisons. Proportion of missing data was inversely associated with coverage estimates in both short (2 years) and longer (5 years) recall of the DHS (*r*=−0.3, *p*=0.02 and *r*=−0.4, *p*=0.004, respectively). The amount of missing information was increased for longer recall compared with shorter recall for all indicators (significant odds ratios ranging between 1.44 and 7.43).

**Conclusions:**

In a context where most LMICs are dependent on population-based household surveys to derive coverage estimates, users of these types of data need to ensure that variability in recall periods and the proportion of missing data across data sources are appropriately accounted for when trend analyses are conducted.

## Introduction

Most sub-Saharan African countries lack robust health system structures for collecting and storing routine health information collected from hospitals, clinics and other health service providers to provide a comprehensive database of individual health histories. At present, coverage estimates of reproductive, maternal, newborn, child and adolescent health and nutrition interventions are mostly obtained using nationally representative household surveys to determine the health status of countries at the national and regional levels. For example, population-based household surveys, including the United States Agency for International Development (USAID)-supported Demographic Health Surveys (DHS) (www.dhsprogram.com/) and the United Nations Children's Fund (UNICEF)-supported Multiple-Indicator Cluster Surveys (MICS) (www.childinfo.org/mics.html), have been used to monitor progress towards the 2015 Millennium Development Goals, particularly those related to maternal and child survival, in low- and middle-income countries (LMICs) (1, 2).

There is collaboration between the DHS and MICS teams through interagency processes to ensure that their survey tools are consistent and similar as far as possible (2). In addition, both surveys adhere to the fundamentals of scientific sampling which include updating sampling frames and calculation of appropriate sample sizes. Therefore, these two survey types can be used together in certain situations to assess coverage of maternal and child health (MCH) indicators. One such scenario would be in combining point estimates from the DHS and MICS to assess how coverage of an indicator has changed over a number of years in a specific area or country. Point estimates from as many time intervals as possible would be needed to obtain a robust analysis of the trends in coverage, hence the need to consider both the MICS and DHS. It has to be noted however that the reference periods used to measure coverage can differ; for example, for antenatal and newborn health indicators, MICS uses births within 2 years preceding the survey as the denominator, whereas the DHS captures data of births within the last 5 years, the latter potentially resulting in greater recall problems (2). This difference becomes critical when coverage trends are assessed using both survey types.

A recent collection of articles focused on issues pertinent to measurement of intervention coverage using DHS and MICS; however, differences in the recall periods between these data sources and the potential effect on trend analyses were not a major focus (3). Some of these studies have highlighted the importance of considering the difference in the length of survey periods covered by the DHS and MICS, but the specific problem of recall bias has not been investigated extensively. One of these studies, a review, highlights, although without confirmatory analyses, that using fewer and most recent years for recall can improve accuracy of information but the sample size could be small [thus widening confidence intervals (CIs)], whereas using a longer recall period might improve the sample size (2). As pointed out by some of these studies, recall bias is generally expected to affect the number of analysable responses in a questionnaire such that even though the sample size may be larger for wider recall periods, there is a high likelihood of underestimating the outcomes due to a high number of ‘missing/no responses or don't know’ and a much larger denominator (4). The study which focuses on information bias in more detail in the collection gives specific practical suggestions to reduce information bias at the data collection stage and not at data analysis stage, although it does acknowledge the need to adjust for it where possible (5).

Much older studies have considered the issue of recall. An assessment of mother's recall of child's vaccination status in Sudan (1989), for example, reported good agreements of between 61 and 81% between mother's recall and records on the vaccination card (6). However, this study only looked at short recall time (<18 months) and was biased towards illiterate women who could not read the vaccination card and naturally relied on their recall. Another old study also found good recall for vaccination of children under the age of 3 years (7). Although the recall for vaccination was very good, because of the short recall period, there was a tendency for error in recalling the exact number of doses and the error increased with fewer doses received. This latter finding indicated that recall bias should be expected for more detailed and complex information.

The issue of the different survey period length (5 years in DHS and 2 years in MICS) for some indicators therefore has not been scrutinised extensively for situations where data or point estimates from both the MICS and DHS need to be analysed collectively. Using point estimates from data collected over different length recall periods is not statistically acceptable. In this study, we asked the following question: Does not adjusting for length of recall period during trend analysis affect coverage trend estimates? DHS and MICS from six sub-Saharan African countries were used to highlight differences in recall periods between the two surveys which can affect coverage trend estimates when point estimates from both sources are used to construct trends. We also investigated how recall period affects the generally known relationship between proportion of missing data or sample size and coverage point estimates.

## Methods

### Coverage indicators and data sources

All available DHS and MICS undertaken in the period 1998–2012 in six African countries, namely Ethiopia, Ghana, Malawi, Mali, Mozambique and Niger, were included in this analysis. Four MCH indicators were used: 1) malaria prevention during pregnancy, 2) vaccination against tetanus during pregnancy, 3) early initiation of breastfeeding and 4) postnatal care for mothers after delivery (Table 1). These were collected retrospectively over a period of 2 and 5 years in MICS and DHS, respectively. The available DHS and MICS included for each country are shown in Table 2. Raw data from all of the available DHS and MICS were used to generate point estimates and 95% CIs for the indicators defined in Table 1 for both survey types. Appropriate sampling weights were used based on the sampling procedure of each survey.

**Table 1:** Definitions for the maternal and child health indicators

Indicator	Recommended definition
Early initiation of breastfeeding	Proportion of newborns put to the breast within 1 hr of birth
IPT	Proportion of pregnant women receiving at least two doses of sulfadoxine/pyrimethamine SP for intermittent preventive treatment in pregnancy (IPTp) for malaria during their last live birth
Postnatal care	Proportion of mothers who received postnatal care within 2 days of delivery (excluding the first hour)
Tetanus toxoid vaccination	Proportion of women receiving two doses of tetanus toxoid vaccination during their last live birth

**Table 2:** DHS and MICS data which were included in the analysis

Country	Available surveys
Ethiopia	DHS 2000, DHS 2005, DHS 2011
Ghana	DHS 1998, DHS 2003, MICS 2006, DHS 2008, MICS 2011
Malawi	DHS 2000, MICS 2006, DHS 2010
Mali	DHS 2001, DHS 2006, MICS 2010
Mozambique	DHS 1997, DHS 2003, MICS 2008, DHS 2011
Niger	DHS 1998, MICS 2000, DHS 2006, DHS 2012

DHS, Demographic and Health Surveys; MICS, Multiple Indicator Cluster Surveys.

### Effect of recall time on calculating coverage trends

Trend analyses were conducted for two scenarios: 1) trend analysis combining point estimates from the standard 5-year DHS recall and the standard MICS with 2-year recall period, hereafter referred to as the ‘unmatched trend’, and 2) trend analysis combining point estimates from the DHS adjusted to 2 years (we restricted analysis to women who had given birth in the past 2 years) and point estimates from the standard MICS 2-year recall, hereafter referred to as the ‘matched trend’. This resulted in 20 unmatched trend tests and 20 matched trend tests (Table 3) (i.e. five countries by four interventions). The Ethiopia data sets were excluded from this analysis because they had no MICS surveys. The ptrend function in STATA was used (8). For all the trend analyses, we used the estimated slope for time effect and its test for non-linear association. We therefore report 95% CIs for the slope and used these to test for differences in slopes between matched and unmatched data and also compare *p*-values for departure from linear trend.

**Table 3:** Comparison of trend results from combined DHS and MICS data using unmatched DHS versus matched DHS

	MICS and unmatched-DHS recall period			MICS and matched-DHS recall period		
		
	Slope (95% CI)	*p*-value for slope	*p*-value for linearity	Slope (95% CI)	*p*-value for slope	*p*-value for linearity
IPT						
Ghana	0.127 (0.116, 0.138)	<0.0001	<0.0001	0.118 (0.106, 0.131)	<0.0001	<0.0001
Malawi	0.078 (0.066, 0.90)	<0.0001	0.691	0.087 (0.072, 0.102)	<0.0001	0.921
Mali	0.130 (0.125, 136)	<0.0001	0.009	0.116 (0.109, 0.123)*	<0.0001	<0.0001
Mozambique	−0.03 (−0.047, −0.019)	<0.0001	#	−0.046 (−0.06, −0.030)	<0.0001	**#**
Niger	0.375 (0.361, 0.389)	<0.0001	#	0.370 (0.353, 0.387)	<0.0001	#
Tetanus toxoid						
Ghana	−0.004 (−0.013, 0.004)	0.001	0.0006	−0.005 (−0.014, 0.003)	0.001	0.0007
Malawi	0.01 (−0.002, 0.021)	0.195	0.399	0.016 (0.002, 0.031)	0.015†	0.053
Mali	0.041 (0.035, 0.047)	<0.0001	<0.0001	0.023 (0.015, 0.030)*	<0.0001	<0.0001
Mozambique	0.013 (0.009, 0.017)	<0.0001	<0.0001	0.011 (0.006, 0.016)	<0.0001	<0.0001
Niger	0.056 (0.051, 0.061)	<0.0001	<0.0001	0.059 (0.054, 0.065)	<0.0001	<0.0001
Early breastfeeding						
Ghana	0.025 (0.018, 0.033)	<0.0001	<0.0001	0.021 (0.012, 0.030)	<0.0001	<0.0001
Malawi	0.016 (0.005, 0.027)	<0.0001	<0.0001	0.006 (−0.008, 0.019)	<0.0001	<0.0001
Mali	0.070 (0.064, 0.076)	<0.0001	<0.0001	0.038 (0.031, 0.045)*	<0.0001	<0.0001
Mozambique	−0.018 (−0.02, −0.015)	<0.0001	0.001	0.006 (0.001, 0.011)*	<0.0001	<0.0001
Niger	0.045 (0.037, 0.052)	<0.0001	<0.0001	0.075 (0.067, 0.083)*	<0.0001	0.054$
Postnatal care						
Ghana	−0.036 (−0.044, −0.028)	<0.0001	0.0001	−0.033 (−0.042, −0.024)	<0.0001	0.0003
Malawi	0.122 (0.113, 0.132)	<0.0001	0.117	0.120 (0.108, 0.132)	<0.0001	0.238
Mali	##			##		
Mozambique	##			##		
Niger	−0.01 (−0.02, −0.0002)	0.047	#	0.076 (0.060, 0.092)*	<0.0001	#

# not enough data points to give estimate result;

## test not done – only one data point available; In comparison of using matched DHS versus unmatched DHS,

† indicates difference in significance of the trend, that is, *p*-value of slope;

* indicates difference in magnitude of the trend, that is, 95% CI of the slope;

$ indicates difference in linearity of the trend; boldface indicates difference in the direction of the trend.

### The effect of recall time on the relationship between missing data or sample size and point estimates

The effect of recall period on the relationship between missing data or sample size and point estimates was evaluated using the DHS data alone for a more accurate comparison between recent and distant recall. Sample sizes and the proportion of missing data between the most recent births (within the past 2 years, shorter recall time) and more distant births (4–5 years prior to the survey, longer recall time) were calculated. Similarly, coverage point estimates were calculated separately for the most recent births (shorter recall) and more distant births (longer recall). The paired Wilcoxon test was used to test whether 1) the median proportion of missing data and 2) the median sample size were significantly different (*p*<0.05) between the longer recall period and the shorter recall period. Spearman's correlation was used to test for an association between point estimates and 1) the proportion of missing data and 2) sample size for each of the two recall periods. Logistic regression was used to assess the effect of recall period on the magnitude of missing data, that is, how distant recall affects missing data compared with recent recall in general. Adjustment for different survey countries and survey years was made in the regression model for each indicator.

## Results

### Effect of recall period on trend estimates

Table 3 presents results of the 18 (two had only one data point and hence could not be tested) comparisons of trend analyses by country, intervention and the type of analysis (unmatched DHS and matched DHS). The bar graphs of the actual coverage proportions per time point and survey type, with both matched and unmatched DHS data, are given in supplementary file 1 (Supplementary Figure 1). The matched and unmatched tests were compared at four levels. Firstly, the direction (given by the sign for the slope) in which the trend is indicated to have taken place over time, that is, has the coverage been increasing or decreasing. Secondly, whether the magnitude of the slope (change in coverage proportion per time period) is significantly different between the two cases, whether it is increasing or decreasing faster in one case compared with the other. Thirdly, whether the change in coverage itself is being indicated as significant or not using the *p*-value for the slope. Lastly, to compare whether both data sets indicate the same type of trend in terms of linearity over time, using *p*<0.05 for a non-linear change.

The matched and unmatched data gave inconsistent interpretations of change in coverage over time for 39% (7/18) of the comparisons (Table 3, values indicated with †, * and $). We found completely different results in two cases: early breastfeeding for Mozambique and postnatal care for Niger (Table 3, values in boldface). Opposing trends were obtained in both cases where the unmatched DHS resulted in a negative trend, implying that coverage of early initiation of breastfeeding and postnatal care decreased with time, whereas the matched DHS resulted in a positive trend for both indicators. For the rest of the data, the directions of change were in agreement. However, non-overlapping CIs for the slope were observed in another four cases (in addition to the two above) where using unmatched DHS implied faster change in coverage for three cases (intermittent preventive treatment (IPT), tetanus toxoid and early breastfeeding in Mali) and slower change for one case (early breastfeeding in Niger) in comparison to matched DHS. There was only one case, tetanus toxoid in Malawi, where the change in coverage was indicated as not significant with unmatched DHS (*p*=0.195 for slope) but significant with matched DHS (*p*=0.015 for slope). The coverage trend in one set of tests (early breastfeeding in Niger) was implied to be linear in one data set but not the other, that is, *p*>0.05 in matched data set and *p*<0.0001 in the unmatched data set. The remaining 61% (11/18) of comparisons gave results with the agreeing interpretations of change in coverage over time.

### The effect of recall period on the relationship between missing data or sample size and coverage point estimates

All four indicators which were collected over a retrospective period of 5 years in the DHS surveys relied solely on mother's recall and no clinic-registered records or clinic cards were used to confirm verbally reported information (see Ghana DHS 2008 questionnaires as an example) (9). To assess if the use of a longer recall period contributed to recall bias, we compared missing data and sample sizes in the DHS data sets between the age groups of 0–23 months (the most recent 2 years) and 37–59 months (the earliest 2 years) within the 5-year survey period used in the DHS (Table 4). The missing data were consisted of missing entries and responses where the interviewee confirmed not knowing the answer, that is, the ‘don't know’ response option on the questionnaire.

**Table 4:** Sample size, point estimates and the proportion of missing data from the most recent 2 years and earliest 2 years of the DHS surveys

	Country and year of DHS survey	Past 0–23 months			Past 37–59 months		
		
Indicator		Point estimates% (CI)	% missing data (CI)	Sample size	Point estimates% (CI)	% missing data (CI)	Sample size
IPT	Ethiopia 2005	2.0 (1.6 to 2.4)	3.4 (2.9 to 3.9)*	4,469	0.8 (0.5 to 1.1)	67 (66 to 68)*	4,414
	Ethiopia 2000	1.0 (0.7 to 1.2)	0.2 (0.1 to 0.4)	4,600	0.8 (0.4 to 1.3)	0.1 (−0.05 to 0.3)	1,502
	Ghana 2008	39.8 (33.4 to 46.6)	3.8 (1.5 to 6.1)	276	28.9 (20.2 to 37.6)	5.9 (1.4 to 10.4)	108
	Ghana 2003	0.9 (0.3 to 2.2)	7.6 (4.8 to 10.8)	344	1.8 (−0.5 to 4.1)	7.5 (2.9 to 12.1)	132
	Malawi 2010	56 (55 to 58)	0.2 (−0.2 to 0.7)	735	62 (55 to 69)	0	277
	Malawi 2000	28 (26 to 31)	36 (33 to 38)	1,909	34 (29 to 40)	36 (31 to 41)	457
	Mali 2001	1.0 (0.7 to 1.3)	0.5 (0.3 to 0.8)	4,449	0.9 (0.3 to 1.5)	0.5 (0.05 to 0.8)	1,119
	Mozambique 2011	37 (36 to 39)	1.0 (0.7 to 1.3)*	4,913	43 (41 to 46)	2.0 (1.3 to 2.8)*	1,345
	Niger 2012	57 (56 to 59)	3.6 (3.1 to 4.1)*	5,332	12 (11 to 13)	78 (77 to 80)*	4,418
	Niger 2006	0.5 (0.3 to 0.7)	0.3 (0.1 to 0.4)	3,918	0	0.4 (0.01 to 0.9)	922
Tetanus toxoid	Ethiopia 2011	31 (29 to 32)	1.1 (0.8 to 1.4)*	4,453	40 (38 to 42)	2.5 (1.8 to 3.3)*	1,782
	Ethiopia 2005	28 (27 to 29)	0.7 (0.4 to 0.9)	4,321	28 (26 to 31)	2.3 (1.5 to 3.1)	1,449
	Ethiopia 2000	15 (14 to 16)	0.8 (0.6 to 1.1)	4,600	20 (18 to 22)	1.3 (0.8 to 1.9)	1,502
	Ghana 2008	51.3 (44.6 to 57.9)	1.7 (0.2 to 3.3)	276	60.9 (51.6 to 70.3)	4.8 (0.7 to 9.0)	108
	Ghana 2003	46.7 (41.2 to 52.2)	1.7 (0.4 to 3.1)	344	49.3 (40.6 to 57.9)	3.7 (0.4 to 7.0)	132
	Ghana 1998	52.5 (45.3 to 57.7)	0.8 (−0.4 to 2.1)	203	57.5 (47.8 to 67.3)	1.9 (−0.8 to 4.5)	102
	Malawi 2010	66 (62 to 70)	0.3 (0 to 0.7)	823	69 (62 to 76)	0	298
	Malawi 2000	58 (56 to 61)	0	1,901	67 (62 to 72)	0	454
	Mali 2001	27 (26 to 28)	1.0 (0.7 to 1.3)	4,449	33 (30 to 36)	1.5 (0.8 to 2.2)	1,119
	Mozambique 2011	53 (52 to 55)	0.9 (0.6 to 1.2)*	4,913	59 (57 to 62)	2.9 (2.0 to 3.8)*	1,345
	Mozambique 2003	57 (55 to 58)	2.0 (1.6 to 2.5)*	4,245	56 (54 to 59)	4.4 (3.3 to 5.5)*	1,379
	Mozambique 1997	49 (48 to 51)	1.9 (1.5 to 2.2)*	5,671	56 (54 to 59)	4.4 (3.3 to 5.5)*	1,379
	Niger 2012	50 (49 to 52)	0.1 (0.02 to 0.2)	5,140	44 (41 to 47)	0.2 (−0.08 to 0.5)	956
	Niger 2006	23 (21 to 24)	0.2 (0.1 to 0.4)	3,918	21 (19 to 24)	0.8 (0.2 to 1.4)	922
Early breastfeeding	Ethiopia 2011	52 (50 to 53)	2.6 (2.1 to 3.0)	4,453	50 (48 to 52)	2.4 (1.7 to 3.1)	1,782
	Ethiopia 2005	66 (65 to 68)	2.8 (2.3 to 3.3)	4,321	67 (64 to 69)	3.7 (2.7 to 4.6)	1,449
	Ethiopia 2000	47 (46 to 49)	2.9 (2.5 to 3.4)	4,600	51 (49 to 54)	2.4 (1.7 to 3.2)	1,502
	Ghana 2008	55.4 (47.5 to 63.1)	1.8 (0.2 to 3.4)	276	53.6 (44 to 63.2)	4.0 (0.2 to 7.7)	108
	Ghana 2003	58.1 (52.3 to 63.7)	1.3 (0.08 to 2.5)	344	62.7 (54.4 to 71.1)	3.1 (0.1 to 6.1)	132
	Ghana 1998	20.5 (15.4 to 26.7)	1.5 (−0.2 to 3.2)	203	13.3 (6.6 to 19.9)	1.0 (−0.9 to 3.0)	102
	Malawi 2010	94 (92 to 96)	1.0 (0.3 to 1.8)	825	98 (96 to 100)	0.5 (−0.3 to 1.2)	298
	Malawi 2000	68 (66 to 71)	0.07 (−0.05 to 0.2)	1,909	67 (62 to 72)	0	457
	Mali 2001	30 (29 to 32)	2.5 (2.1 to 3.0)	4,449	29 (26 to 32)	3.4 (2.3 to 4.4)	1,119
	Mozambique 2011	74 (73 to 75)	6.0 (5.4 to 6.7)*	5,085	23 (22 to 25)	70 (68 to 71)*	4,248
	Mozambique 2003	64 (62 to 65)	1.5 (1.2 to 1.9)	4,245	60 (57 to 63)	1.4 (0.8 to 2.1)	1,379
	Mozambique 1997	68 (67 to 69)	1.5 (1.2 to 1.8)	5,671	60 (57 to 63)	1.4 (0.8 to 2.1)	1,379
	Niger 2012	61 (60 to 62)	0.1 (0.04 to 0.2)*	6,567	48 (45 to 51)	6.9 (5.3 to 8.4)*	1,027
	Niger 2006	47 (45 to 58)	1.3 (0.9 to 1.6)*	3,918	47 (44 to 50)	2.6 (1.6 to 3.7)*	922
Postnatal care	Ethiopia 2011	6.7 (6.0 to 7.4)	91 (90 to 92)	4,453	7.4 (6.2 to 8.6)	91 (89 to 92)	1,782
	Ethiopia 2005	4.3 (3.7 to 4.9)	94 (94 to 95)	4,321	6.3 (5.1 to 7.6)	93 (92 to 95)	1,449
	Ethiopia 2000	2.7 (2.3 to 3.2)	0.2 (0.04 to 0.3)	4,600	4.8 (3.7 to 5.9)	0.1 (−0.05 to 0.3)	1,502
	Ghana 2008	6.2 (4.0 to 9.6)	51.5 (45.6 to 57.5)	276	13.1 (6.7 to 19.6)	55.1 (45.5 to 64.6)	108
	Ghana 2003	14.8 (10.9 to 19.6)	71.4 (66.6 to 76.2)	344	11.6 (6.1 to 17.2)	74.2 (66.7 to 81.8)	132
	Ghana 1998	26.2 (19.4 to 34.4)	46.6 (39.7 to 53.5)	203	27.6 (18.8 to 36.4)	39.2 (29.5 to 48.8)	102
	Malawi 2010	41 (38 to 45)	51 (46 to 55)	825	44 (37 to 51)	44 (37 to 51)	298
	Malawi 2000	3 (2 to 3)	94 (93 to 95)	1,909	2 (0.4 to 4.4)	96 (95 to 97)	457
	Mali 2001	7.5 (6.8 to 8.3)	90 (89 to 91)	4,449	9.6 (7.8 to 11.3)	88 (86 to 90)	1,119
	Mozambique 2003	5.3 (4.6 to 5.0)	81 (79 to 82)	4,245	6.8 (5.4 to 8.1)	80 (78 to 82)	1,379
	Niger 2012	16 (15 to 17)	44 (42 to 45)*	4,093	15 (14 to 16)	77 (75 to 78)*	3,889
	Niger 2006	12 (11 to 13)	79 (78 to 81)	3,190	13 (10 to 15)	81 (78 to 83)	763

* No overlap in confidence intervals of missing data between the last 2-year estimate and the earliest 2-year estimate.

The overall paired test to compare the two recall periods indicated a very strong difference in the median sample sizes (*p*<0.0001). However, sample size was not associated with point estimates overall, nor for distant or recent recalls separately (Table 5: distant recall rho=−0.25 and *p*=0.07; recent recall rho=−0.04 and *p*=0.78; overall rho=−0.1 and *p*=0.30). The point estimates for the two recall periods in DHS are presented in Table 4. A direct comparison of point estimates between distant and recent recall was not made for one important reason: It is possible that coverage increases over time and hence can cause the most recent recall time to have higher point estimates. It was therefore not possible to adjust for this inherent bias between the two groups in order to make a more accurate comparison.

**Table 5:** The relationship between proportion of missing data and coverage estimates or sample size

Spearman Correlation	Spearman's rho	*p*
Coverage estimates:		
All data	−0.36	0.0002
0–23 months	−0.32	0.02
37–59 months	−0.40	0.004
Sample size:		
All data	−0.10	0.30
0–23 months	0.04	0.78
37–59 months	−0.25	0.07

The proportion of missing data was found to be higher in the data collected through recall from the earliest 2 years than that collected from recalling information over the most recent 2 years, for 11 comparisons (Table 4). Missing data for tetanus toxoid vaccination uptake during pregnancy were higher for recall from the earliest 2 years in all three Mozambique DHS and the Ethiopia 2011 DHS. A similar pattern was observed for early initiation of breastfeeding in one Mozambique survey and two Niger surveys. Similarly, missing data for IPT were greater in the earliest 2 years for one survey in Ethiopia, Mozambique and Niger. Only one such difference was seen in the postnatal care data from the Niger 2012 DHS. An overall paired Wilcoxon test indicated a significant difference in the medians of missing data proportions for all indicators between longer recall and shorter recall (*p*=0.003). The median of missing data proportions was higher for longer recall time (3.3%) and lower for shorter recall time (1.7%).

**Fig. 1:**
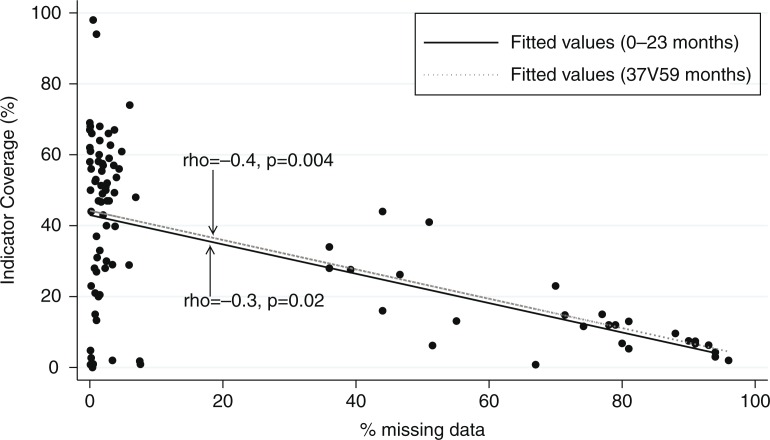
The relationship between the proportion of missing data and observed point estimates. The relationship between the proportion of missing data and observed point estimates for the four indicators for data recalled from the 2 years and 4 to 5 years prior survey date.

There was a significant negative correlation between the amount of missing data and the coverage levels for the four indicators. The correlation was slightly stronger for longer recall time alone (Spearman's correlation rho=−0.4 and *p*=0.004 compared to rho=−0.3 and *p*=0.02 in most recent recall) (Fig. 1, Table 5). Using the logistic regression model and adjusting for survey country and year, distant recall was associated with higher amounts of missing data compared with recent recall for each of the four indicators [Table 6: IPT OR=5.43 (5.13,5.76); Tetanus toxoid OR=6.77(6.39,7.16); early breastfeeding OR=5.54(5.24,5.84); postnatal care OR=1.85(1.75,1.95)].

**Table 6:** The effect of recall period on amount of missing data for each indicator

Indicator	Odds ratio (95% CI) 37–59 months versus 0–23 months	*p*
IPT	5.43 (5.13, 5.76)	<0.0001
Tetanus toxoid	6.77 (6.39, 7.16)	<0.0001
Early breastfeeding	5.54 (5.24, 5.84)	<0.0001
Postnatal care	1.85 (1.75, 1.95)	<0.0001

The regression analyses were adjusted for survey year and country for each indicator.

### Implications of changing indicator definitions on coverage trend analysis

There are some indicators whose current recommended definition for evaluating change could not be generated from earlier surveys, making comparison across surveys less reliable. Older survey questionnaires in some cases did not provide certain information needed to generate indicators as currently recommended (1). The most common problem was obtaining data for postnatal care during the first 2 days with exclusion of the first hour following delivery. No hourly information was available in at least one survey from all countries except Ethiopia. The other common problems were lack of IPT dosage counts. If older surveys need to be included in an analysis, it may be necessary to redefine indicators less specifically, to allow for the inclusion of as many data points as possible to provide sufficient power to demonstrate trends in coverage. In the case of IPT, for example, the definition could be simplified from ‘2 doses of tetanus toxoid vaccine within the appropriate interval prior to the infant's birth’ to just ‘received a dose’. The first hour following birth can also be included within 48 hr after delivery, in the case of postnatal care rather than being excluded in some point estimates and not others in the same trend test.

## Discussion

We have shown that combining data over multiple survey years, without matching the recall period over which the data were collected, can lead to inaccurate estimates of coverage trends and therefore incorrect inferences about trends in MCH intervention coverage. Also, we have highlighted that longer recall increases the likelihood of having high rates of missing information during survey data collection and missing data tend to bias coverage estimates. The magnitude of these effects can vary from one setting to another.

Here, the recall period for DHS was reduced from 5 to 2 years to match the MICS when performing quantitative coverage trend analyses. Our example shows that using the standard 5-year recall period for DHS rather than matching it to the 2-year recall period for MICS gave conflicting interpretations of coverage trends over time for almost half of the data, with the largest discrepancy being opposing trends for early breastfeeding in Mozambique and postnatal care in Niger. Using unmatched recall periods would have falsely indicated a strong decline in coverage for these two health indicators although the opposite was true. The fact that the two approaches, matching and not matching recall times, yield different results in 39% of the tests highlights the need to use consistent recall periods for generating point estimates. Our results show that failure to match recall periods does indeed affect the accuracy of reported coverage trends. The practical steps suggested by Cutts and colleagues on how to reduce information bias at the data collection level need to be coupled with adjustments at the data analyses level (5). Their suggested steps could be implemented in future survey data collection, but the current survey data already available require more scrutiny at the analyses level. We have presented some of the major considerations at analysis level. These recommended considerations and adjustments are not restricted to MICS and DHS alone but are applicable to any study which uses different survey types and retrospective data.

Using data from verbal responses alone is likely to be affected by failure to recall information over longer time periods. Therefore, the likelihood of having missing data and ‘don't know’ responses is increased as retrospective recall time increases. The effect of information bias due to reliance on verbal responses alone has been discussed previously but no detailed assessment of the impact of the length of recall period has been reported (6, 7, 9, 10). We have shown here that data from a longer retrospective time reduce the amount of valuable responses which increases the amount of missing information. As already known, the proportion of missing information is known to have a negative correlation with point estimates. The magnitude of these relationships, as shown here, can vary from one setting to another; hence, assumptions should not be made based on results from a different setting.

The strongest effect observed in the analyses was underestimation of the point estimates as a result of a higher proportion of missing data, and this negative relationship was larger when recall time was more than 2 years in the past. Therefore, we have confirmed that recall time is an effect modifier of the relationship between point estimates and proportion of missing data. Sample size was not an issue in these data as it did not influence the coverage estimates. It is known, and has been discussed in other literature, that poor sample size reduces the precision of the point estimates and therefore even though it was not a problem in this data set, it is an important consideration for all survey data analyses (4). This finding further confirms that the effect of missing data is not the result of underlying sample size problems but a clear indication of recall problems with longer retrospective survey time.

It is therefore very important for studies carrying out statistical analyses to first assess whether the amount of missing data influences outcomes and if so, depending on the data, consider appropriate methods for adjusting for missing data. It has to be noted that the length of recall which can introduce bias in an analysis can vary between situations, depending on the complexity of the information being recalled and the interviewee (e.g. literacy levels), such that in other cases time in years becomes an issue, yet in others only time in weeks (11, 12). Therefore, assumptions should not be made about when to consider adjusting for recall, rather data should be assessed *a priori* for the potential of recall bias.

Definitions of key indicators have changed over time within DHS and MICS. Therefore, definitions of indicators need to be investigated during trend analysis to ensure comparability. It may be necessary to modify indicator definitions of more recent surveys to fit definitions of older surveys when doing trend analysis, thus increasing data points and statistical power.

### Limitations

We have purposely used only two survey types in this analysis because they are the most similar and widely used for coverage trend analyses in LMICs. However, it has to be noted that estimates of coverage can also be obtained from other population-based household surveys such as the Malaria Indicator Surveys developed by the Roll Back Malaria Monitoring and Evaluation Reference Group, the WHO-supported Expanded Program on Immunization surveys and the Lot Quality Assurance Surveys, though they may be more limited in scope and serve different purposes (13, 14). Irrespective of the survey type, differences between all surveys used in an analysis need to be accounted for.

There are other underlying differences between MICS and DHS which could not be investigated here. DHS collect information mostly from biological mothers, whereas MICS sample all caretakers in the household; therefore, data include orphans and foster children, regardless of whether the biological mothers are present in the household. These do not affect the results presented here because the four indicators that were analysed ask women who have had children to respond in relation to their last birth and hence do not include foster children or orphans.

Correlation analyses at country level could not be done separately because of the reduced sample sizes which weaken tests and confidence in such results. Alternatively, we adjusted for difference in survey country and survey year in the logistic regression models. We still acknowledge that, given adequate country-level data, the magnitudes of associations between recall, missing data and sample sizes could vary from one country to another depending on data quality and other data collection-related factors.

## Conclusion

The DHS and MICS are a very rich source of MCH data and are important for evaluating changes in coverage over time in LMICs and monitoring progress towards global targets. Though these surveys are largely similar, they do collect data over different recall periods; therefore, users and consumers of these types of data need to ensure that variability in recall periods and the proportion of missing data across data sources are appropriately accounted for when trend analyses are conducted. Similarly, use of different survey types for any kind of analyses generally requires consistency to yield accurate interpretations.

## Supplementary Material

Does adjusting for recall in trend analysis affect coverage estimates for maternal and child health indicators? An analysis of DHS and MICS survey data

## Paper context

Large population-level surveys like the Demographic and Health Survey are rich data sources for evaluating health progress. Assessing trends over time often requires a combination of different types of these data sources. However, for some health indicators, interview recall times during data collection vary between surveys. Here, we highlight the importance of adjusting for varying recall times by showing simplistically that recall times affect the amount of missing data and indicator estimates.
