# Supplementary material for: Does adjusting for recall in trend analysis affect coverage estimates for maternal and child health indicators? An analysis of DHS and MICS survey data
Source: Glob Health Action. 2016 Nov 7;9:10.3402/gha.v9.32408. doi: 10.3402/gha.v9.32408 (PMC5102105; doi:10.3402/gha.v9.32408)
Supplement: Does adjusting for recall in trend analysis affect coverage estimates for maternal and child health indicators? An analysis of DHS and MICS survey data [file GHA-9-32408-s001.pdf]

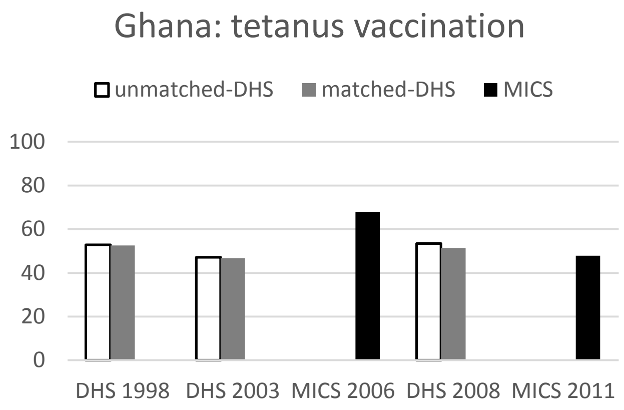
Ghana: tetanus vaccination
unmatched-DHS
matched-DHS
MICS
100
80
60
40
20
0
DHS 1998
DHS 2003
MICS 2006
DHS 2008
MICS 2011

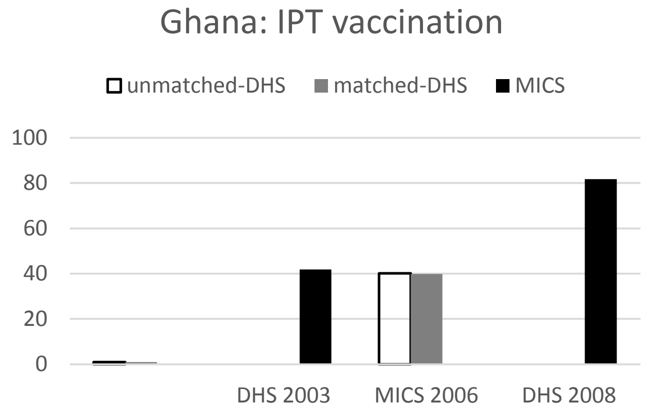
Ghana: IPT vaccination
unmatched-DHS
matched-DHS
MICS
100
80
60
40
20
0
DHS 2003
MICS 2006
DHS 2008

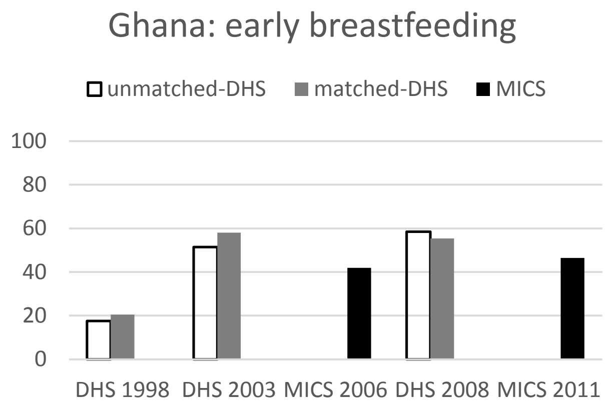
Ghana: early breastfeeding
unmatched-DHS
matched-DHS
MICS
100
80
60
40
20
0
DHS 1998
DHS 2003
MICS 2006
DHS 2008
MICS 2011

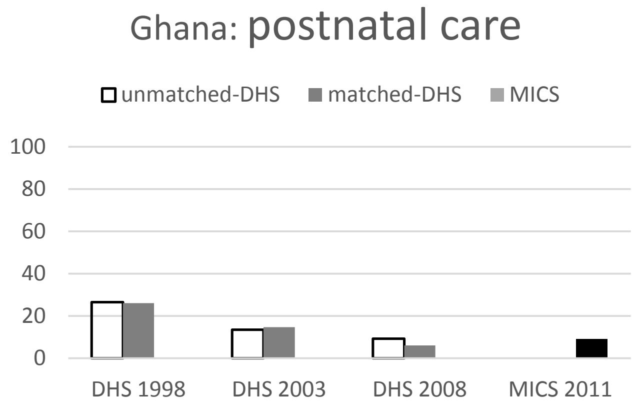
Ghana: postnatal care
unmatched-DHS
matched-DHS
MICS
100
80
60
40
20
0
DHS 1998
DHS 2003
DHS 2008
MICS 2011

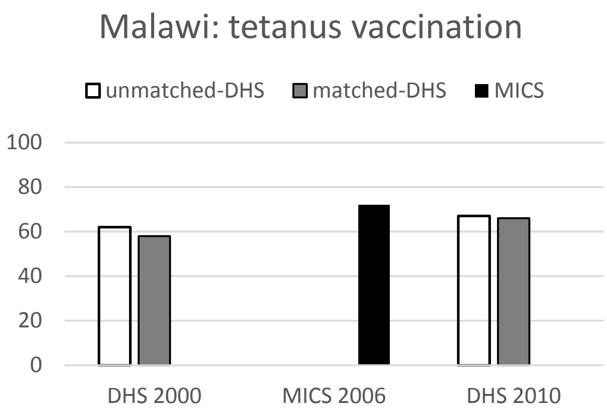
Malawi: tetanus vaccination
unmatched-DHS
matched-DHS
MICS
100
80
60
40
20
0
DHS 2000
MICS 2006
DHS 2010

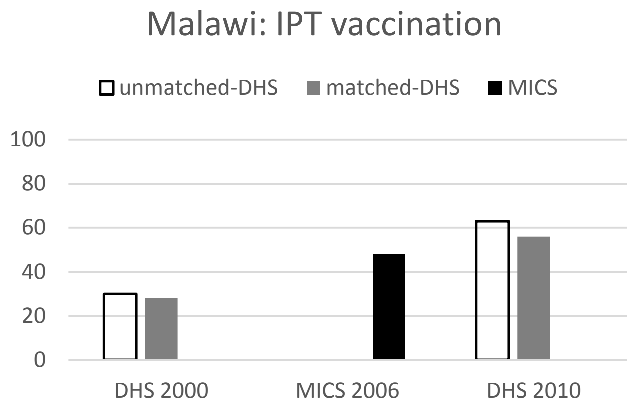
Malawi: IPT vaccination
unmatched-DHS
matched-DHS
MICS
100
80
60
40
20
0
DHS 2000
MICS 2006
DHS 2010

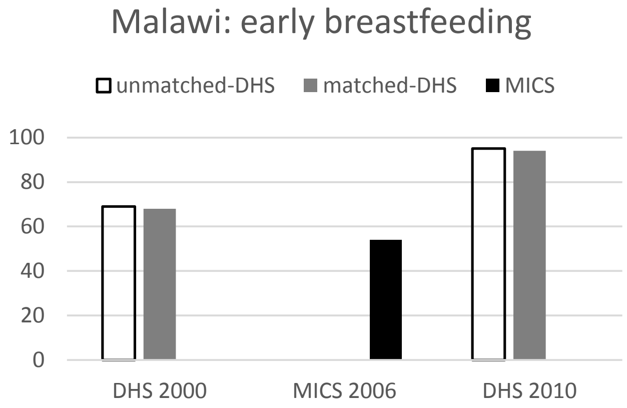
Malawi: early breastfeeding
unmatched-DHS
matched-DHS
MICS
100
80
60
40
20
0
DHS 2000
MICS 2006
DHS 2010

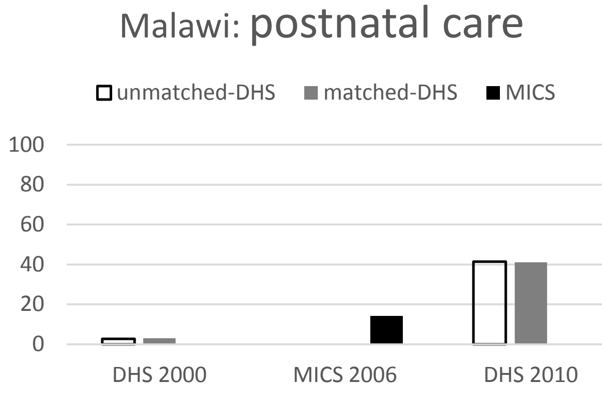
Malawi: postnatal care
unmatched-DHS
matched-DHS
MICS
100
80
60
40
20
0
DHS 2000
MICS 2006
DHS 2010

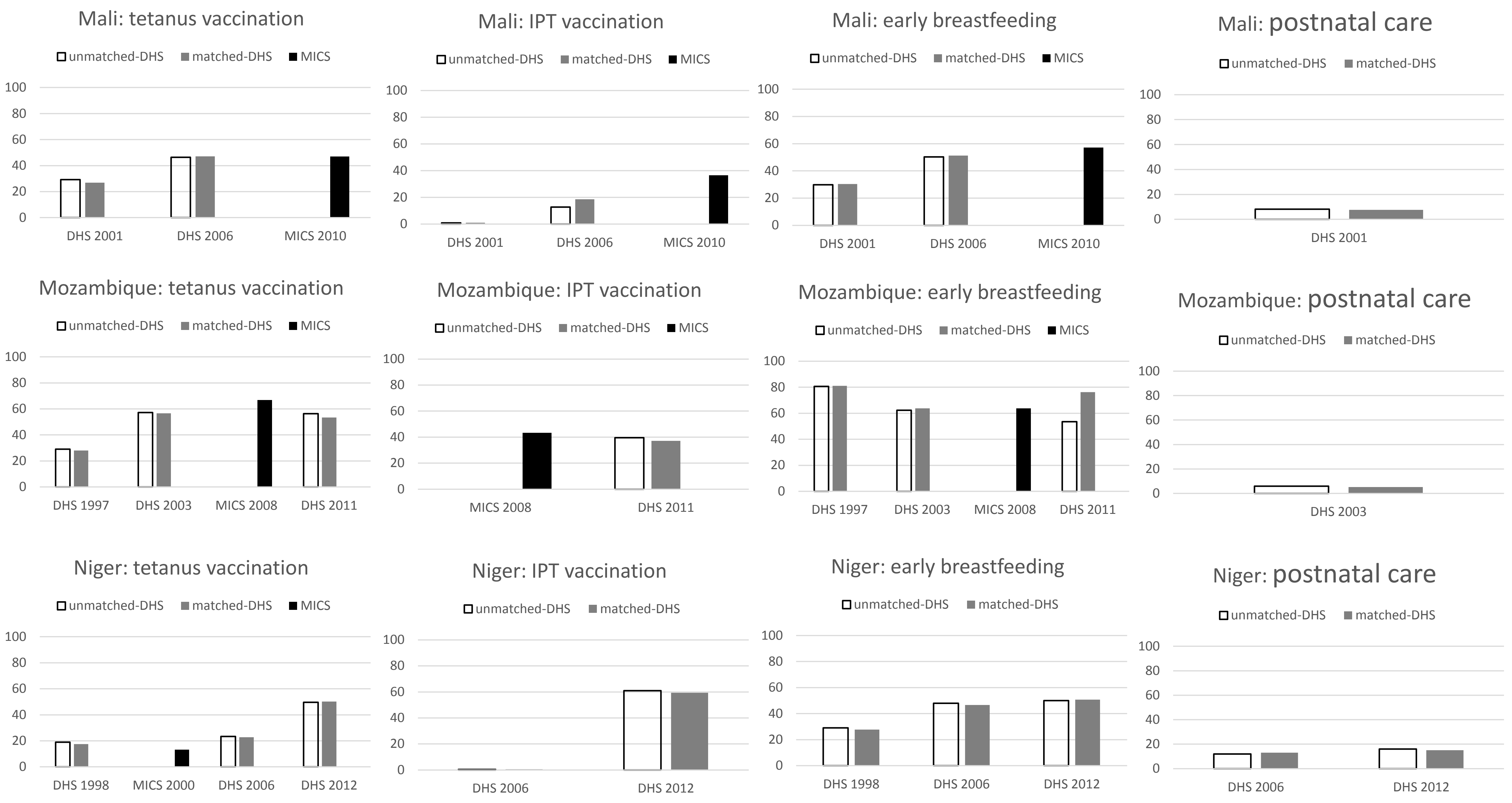
Mali: tetanus vaccination
unmatched-DHS matched-DHS MICS
DHS 2001 DHS 2006 MICS 2010
Mali: IPT vaccination
unmatched-DHS matched-DHS MICS
DHS 2001 DHS 2006 MICS 2010
Mali: early breastfeeding
unmatched-DHS matched-DHS MICS
DHS 2001 DHS 2006 MICS 2010
Mali: postnatal care
unmatched-DHS matched-DHS
DHS 2001
Mozambique: tetanus vaccination
unmatched-DHS matched-DHS MICS
DHS 1997 DHS 2003 MICS 2008 DHS 2011
Mozambique: IPT vaccination
unmatched-DHS matched-DHS MICS
MICS 2008 DHS 2011
Mozambique: early breastfeeding
unmatched-DHS matched-DHS MICS
DHS 1997 DHS 2003 MICS 2008 DHS 2011
Mozambique: postnatal care
unmatched-DHS matched-DHS
DHS 2003
Niger: tetanus vaccination
unmatched-DHS matched-DHS MICS
DHS 1998 MICS 2000 DHS 2006 DHS 2012
Niger: IPT vaccination
unmatched-DHS matched-DHS
DHS 2006 DHS 2012
Niger: early breastfeeding
unmatched-DHS matched-DHS
DHS 1998 DHS 2006 DHS 2012
Niger: postnatal care
unmatched-DHS matched-DHS
DHS 2006 DHS 2012
0 20 40 60 80 100
